# Effectiveness of two-dimensional shear-wave sonoelastography in the diagnosis and follow-up of infantile hypertrophic pyloric stenosis

**DOI:** 10.1007/s00383-024-05738-5

**Published:** 2024-06-25

**Authors:** Sabri Demir, Havva Akmaz Unlu, Gulsah Kiris Uzun, Can Ihsan Oztorun, Ahmet Erturk, Mujdem Nur Azili, Emrah Senel

**Affiliations:** 1 Department of Pediatric Surgery, University of Health Sciences, Ankara Bilkent City Hospital, Children Hospital, Ankara, Turkey; 2 Department of Pediatric Radiology, Ankara Bilkent City Hospital, Children Hospital, Ankara, Turkey; 3 Department of Pediatric Surgery, Ankara Bilkent City Hospital, Children Hospital, Ankara, Turkey; 4 Present Address: Department of Pediatric Surgery, Ankara Yildirim Beyazit University, Faculty of Medicine, Ankara, Turkey

**Keywords:** Infantile hypertrophic pyloric stenosis, Tissue elasticity, Two-dimensional shear-wave sonoelastography

## Abstract

**Introduction:**

We sought to determine the effectiveness and utility of two-dimensional shear-wave sonoelastography (2D-SW-SE) in the diagnosis and postoperative follow-up of infantile hypertrophic pyloric stenosis (IHPS).

**Materials and Methods:**

Twenty-three infants were included in the study, 13 in the IHPS group and 10 in the control group (CG). Preoperative B-mode ultrasonography measurements (longitudinal length and single-wall thickness of the pylorus) and 2D-SW-SE measurements (pylorus tissue stiffness and shear-wave propagation speed) were compared between the groups. The infants with IHPS then underwent Ramstedt pyloromyotomy and were invited for follow-ups on the tenth day and the first, third, and sixth months postoperatively. Measurements taken at the follow-ups were compared with the preoperative values.

**Results:**

No differences were found between the groups regarding age, gender, body weight, or week of birth. The pyloric lengths in the IHPS group were longer than in the CG (p < 0.001), and the single-wall thicknesses were thicker (p < 0.001). The pylorus in the IHPS group was four times stiffer than in the CG (27.4 kPa versus 7.66 kPa), and the shear-wave propagation speed in the tissue was higher (1.34 m/s versus 2.69 m/s; p < 0.001). Both values decreased over time in the IHPS group and were normal by the third postoperative month.

**Conclusions:**

2D-SW-SE can be used as an assistive imaging tool alongside B-mode ultrasound for diagnosing IHPS. It can also be used to identify inadequate surgery by detecting whether the pyloric tissue has softened at follow-up.

## Introduction

Infantile hypertrophic pyloric stenosis (IHPS) is the most common reason for surgical intervention in the first month of life [1]. The pyloric canal narrows and the wall thickness increases due to hyperplasia and hypertrophy of the pyloric muscles, which is accompanied by projectile, non-bilious vomiting due to obstruction of the gastric outlet; this occurs suddenly in previously healthy infants [2, 3], and diagnosis is most commonly made 3–5 weeks after birth [1]. IHPS occurs in 2–4 of every 1000 live births in Caucasians but is less common in other ethnicities, and it is 4–6 times more common in boys than girls [4, 5]. Its etiology is not fully elucidated, but genetic and environmental factors are thought to play a role in its etiopathogenesis [4].

A diagnosis of IHPS is made by palpation of the hypertrophic pylorus on physical examination (olive sign) and/or by B-mode ultrasonography (US) imaging. Although palpation of the pylorus is diagnostic, its detection rate at the time of diagnosis has decreased to 13.6% due to the increasing use of B-mode US [6], which is now the gold standard diagnostic method. A diagnosis is made when the single-wall thickness of the pylorus is greater than 3 mm in transverse measurement and its length is longer than 15 mm longitudinally [7, 8]. Ramstedt pyloromyotomy (open or laparoscopic) has been the treatment of choice since it was described by Ramstedt in 1911 [9].

Elasticity is the ability of tissues to deform with external pressure and return to their original shape when the pressure is removed and ultrasound elastography (USE) is an assistive ultrasound technology used to evaluate the elasticity of tissues [10]. The data obtained are similar to manual palpation but with higher sensitivity, and interest in the technique has steadily increased since it was first introduced in the 1990s, with two techniques currently used: strain elastography (SE) and shear-wave USE (SW-USE) [11, 12]. In SE, the elasticity of the tissues—usually superficial tissues—is evaluated by a compression force applied externally, whereas in SW-USE, short-term, high-power acoustic impulse waves are sent to the tissue with US probes, which cause small displacements in the tissues (1–10 µm). These displacements in the horizontal plane are called “shear waves,” and the velocity of the waves in the tissue is directly proportional to the tissue’s stiffness. The velocity values obtained thus show the objective elasticity of the tissues, with the shear-wave propagation velocity measured in meters per second (m/s) and the stiffness in kilopascals (kPa) [13].

In the two-dimensional shear-wave sonoelastography (2D-SW-SE) technique, the force of sonic radiation displaces the tissue at multiple points, which allows the simultaneous measurement of elasticity at more than one point. In readouts, the tissues are colored according to their hardness—hard tissues in red, soft tissues in blue, and intermediate in green [14]. We hypothesized that 2D-SW-SE used in conjunction with conventional B-mode US in the diagnosis of IHPS and postoperative follow-up would provide higher diagnostic accuracy, and the aim of this study was to test this hypothesis. In a literature search conducted in both English and Turkish, no studies were found regarding the use of USE in the diagnosis of IHPS, and we, therefore, believe that this is the first study on the topic.

## Materials and methods

The study was designed prospectively, and infants who were diagnosed with IHPS and treated in our pediatric surgery clinic between June 2019 and June 2021 were included. The necessary ethical approval was obtained from the local ethics committee (Date: May 9, 2019; No: 2019-132). Patients who underwent surgery for IHPS were enrolled in the IHPS group, and a control group (CG) was formed from healthy infants of similar ages who were treated in the clinic for inguinal hernia repair, circumcision, or umbilical granuloma during the same period. The parents of infants in both groups were given detailed information about the study, and infants were included only after the parents agreed and provided signed informed consent.

### Data description

For all subjects, the single-wall thickness and longitudinal length of the pylorus were measured using gray-scale B-mode US and the tissue stiffness and shear-wave propagation velocity using 2D-SW-SE. For patients who were hospitalized for vomiting and suspected IHPS, the B-mode US was performed preoperatively after physical examination for diagnostic purposes, with 2D-SW-SE measurements made at the same time. The sex, age (in weeks), weeks of birth, and body weight (g) of all subjects were also recorded, together with the duration of vomiting (in days) of the subjects in the IHPS group. Ramstedt pyloromyotomy was then performed with the open technique for all subjects in the IHPS group.

### Postoperative follow-up

The patients were discharged after tolerating oral feeding and were invited for follow-up at 10 days and at 1, 3, and 6 months postoperatively. B-mode US and 2D-SW-SE were performed on patients who returned for follow-up.

### B-mode US and 2D-SW-SE assessments

**Fig. 1:**
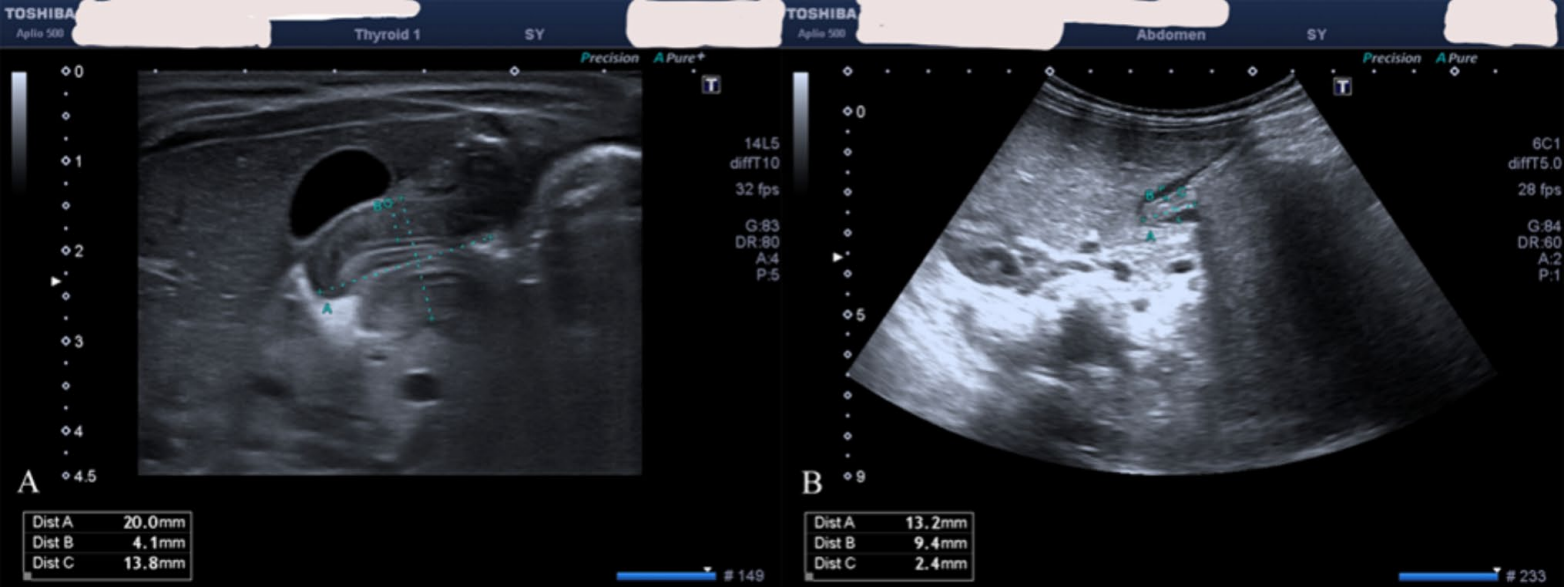
B-Mode US images of IHPS. Patients with a single-wall thickness greater than 3 mm and a longitudinal length greater than 15 mm were diagnosed with IHPS. **A** the single wall thickness of the pylorus of the patient diagnosed with IHPS was 4.1 mm, the double wall thickness was 13.8 mm, and the longitudinal length was measured as 20 mm. **B** in the 3rd month post-operative B-Mode US image of the same patient, it was seen that the longitudinal length decreased to 13.2 mm, the single wall thickness decreased to 2.4 mm, and the double wall thickness decreased to 9.4 mm

All B-mode US and 2D-SW-SE evaluations were performed using a Canon–Toshiba Aplio 500 Platinum US device (Canon Medical Systems Co. Ltd.) with a high-frequency linear probe set to the small-parts preset (frequency range: 5–14 MHz) by the same experienced pediatric radiologist. Patients with a single-wall thickness greater than 3 mm and a longitudinal length greater than 15 mm, as measured by the B-mode US, were diagnosed with IHPS (Fig. 1 A, B).

**Fig. 2.**
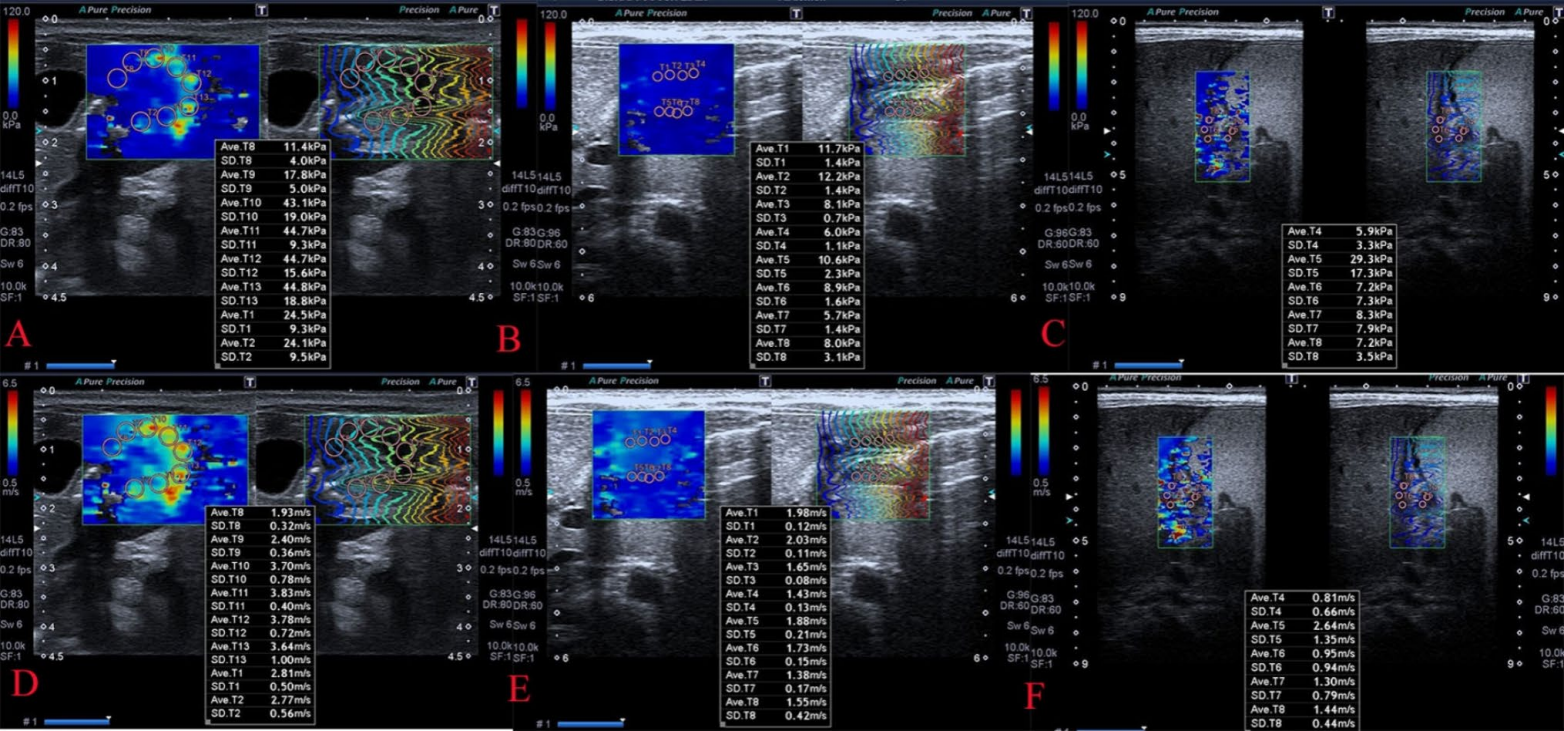
2D-SW-SE images of IHPS. Measurements were taken from 5 to 8 adjacent points to evaluate the pyloric tissue stiffness. The images in the upper row show kPa values and those in the lower row show m/s values. Figure 2**A** and **D**. The patient’s preoperative values. Figure 2**B** and **E**. Postoperative 1st month values are shown. Figure 2**C** and **F**. The values decreased to normal by the 3rd postoperative month, and the pyloric tissue softened

2D-SW-SE measurements of tissue stiffness were made at 5–8 points of the pylorus, and the mean values were calculated. The same evaluations were performed at each follow-up (Fig. 2 A–F) and for the infants in the CG.

### Statistical analysis

Statistical analysis was performed with the Statistical Package for the Social Sciences (SPSS) Software Version 21 (SPSS Inc., Chicago, IL, USA). Numerical variables are expressed as mean ± SD and categorical variables as percentages. Visual and analytical methods were used to investigate whether numerical variables, such as the infants’ ages, wall thickness and length of the pylorus, and 2D-SW-SE measurements, were normally distributed. Shapiro–Wilk tests were used to evaluate normality because the number of infants in the study was less than 50. Descriptive analysis of non-normally distributed numerical variables was performed with Mann–Whitney *U* tests, and Student’s *t*-test was used to analyze the mean values of the normally distributed variables. Chi-squared tests or Fisher’s exact tests were used to compare categorical variables. For all tests, *p* < 0.05 was considered significant.

It was determined that the pyloric length, single-wall thickness, tissue stiffness, and propagation velocity values, both preoperatively and at follow-up, of the infants in the IHPS group did not comply with parametric test assumptions, so change over time for these parameters was examined using Friedman tests. Pairwise comparisons were made using Wilcoxon tests with a Bonferroni correction.

## Results

### Demographic data of patients

Thirteen infants underwent surgery for IHPS from June 2019 to June 2021—twelve (92.3%) male and one female (7.7%) with a mean age of 5.05 ± 1.70 weeks. Ten infants were included in the CG—eight (80%) boys and two girls (20%) with a mean age of 6.06 ± 0.73 weeks. There were no significant differences between the groups in age (*p* = 0.074), sex (*p* = 0.560), body weight (*p* = 0.248), or birth weeks (*p* = 0.373). Demographic data are given in Table 1.

**Table 1:** Demographic data of patients

	IHPS group (*n* = 13)	Control group (*n* = 10)	*p*
Age (Week), mean (± SD)	5.05 (± 1.70)	6.06 (± 0.73)	0.074*
Gender			
Male	12 (92.3%)	8 (80.0%)	0.560**
Female	1 (7.7%)	2 (20.0%)	
Weight (gram), mean (± SD)	3620.00 (± 799.42)	4050.67 (± 936.89)	0.248*
Birth weeks (week), mean (± SD)	39.54 (± 2.00)	38.77 (± 2.02)	0.373*

*Student's *t* test used

**Fisher’s exact test used

### Comparison of B-mode US and 2D-SW-SE measurements between the IHPS group and CG

The mean pyloric duct lengths of the infants in the IHPS group (20.68 mm) were longer than those of the infants in the CG (11.39 mm; *p* < 0.001). The mean single-wall pyloric thickness of the IHPS group (4.74 mm) was significantly greater than that of the CG (2.27 mm; *p* < 0.001).

The pyloric tissues of the infants in the IHPS group (27.4 ± 6.05 kPa) were almost four times harder than those of the CG (7.66 ± 2.73 kPa; *p* < 0.001), and the propagation velocities of the shear waves in the IHPS group (2.69 ± 0.29 m/s) were accordingly double those of the CG (1.34 ± 0.25 m/s; *p* < 0.001). The between-groups comparisons of the B-mode US and 2D-SW-SE measurements are shown in detail in Table 2.

**Table 2:** Comparative statistical analysis (p*) of preoperative B-Mode US and 2D-SW-SE pyloric values of the IHPS group and comparative statistical analysis (p**) of the preoperative, postoperative 10th day, first month, third month, and sixth-month values

Variables	Control group	Preoperative	Postoperative 10th day	Postoperative 1st month	Postoperative 3rd month	Postoperative 6th month	*p* values*	*p* values**
Lenght of pylorus (mm), mean (± SD)	11.39 (± 1.72)	20.68 (± 2.22)	17.62 (± 3.51)	15.73 (± 1.90)	14.36 (± 2.05)	12.58 (± 1.07)	< 0.001^a^	0.005^c^
Single-wall thickness(mm), mean (± SD)	2.27 (± 0.27)	4.74 (± 0.65)	4.32 (± 1.10)	4.01 (± 0.60)	3.24 (± 1.75)	2.36 (± 0.63)	< 0.001^b^	0,056^c^
kPa, mean (± SD)	7.80 (± 2.43)	27.04 (± 6.05)	14.87 (± 4.08)	10.19 (± 4.77)	8.65 (± 1.87)	7.86 (0.55)	< 0.001^b^	0.022^c^
ms, mean (± SD)	1.34 (± 0.22)	2.69 (± 0.29)	1.92 (± 0.36)	1.59 (± 0.20)	1.32 (± 0.22)	1.34 (± 0.25)	< 0.001^b^	0.022^c^

*Preoperative values of the IHPS group and values of the control group were compared

**The preoperative values of the infants in the IHPS group and their values at the postoperative 10th day, first month, third month and sixth month were compared

ªMann–Whitney *U* test used

^b^Student's *t* test used

^c^Friedman test used

### Postoperative results

**Fig. 3:**
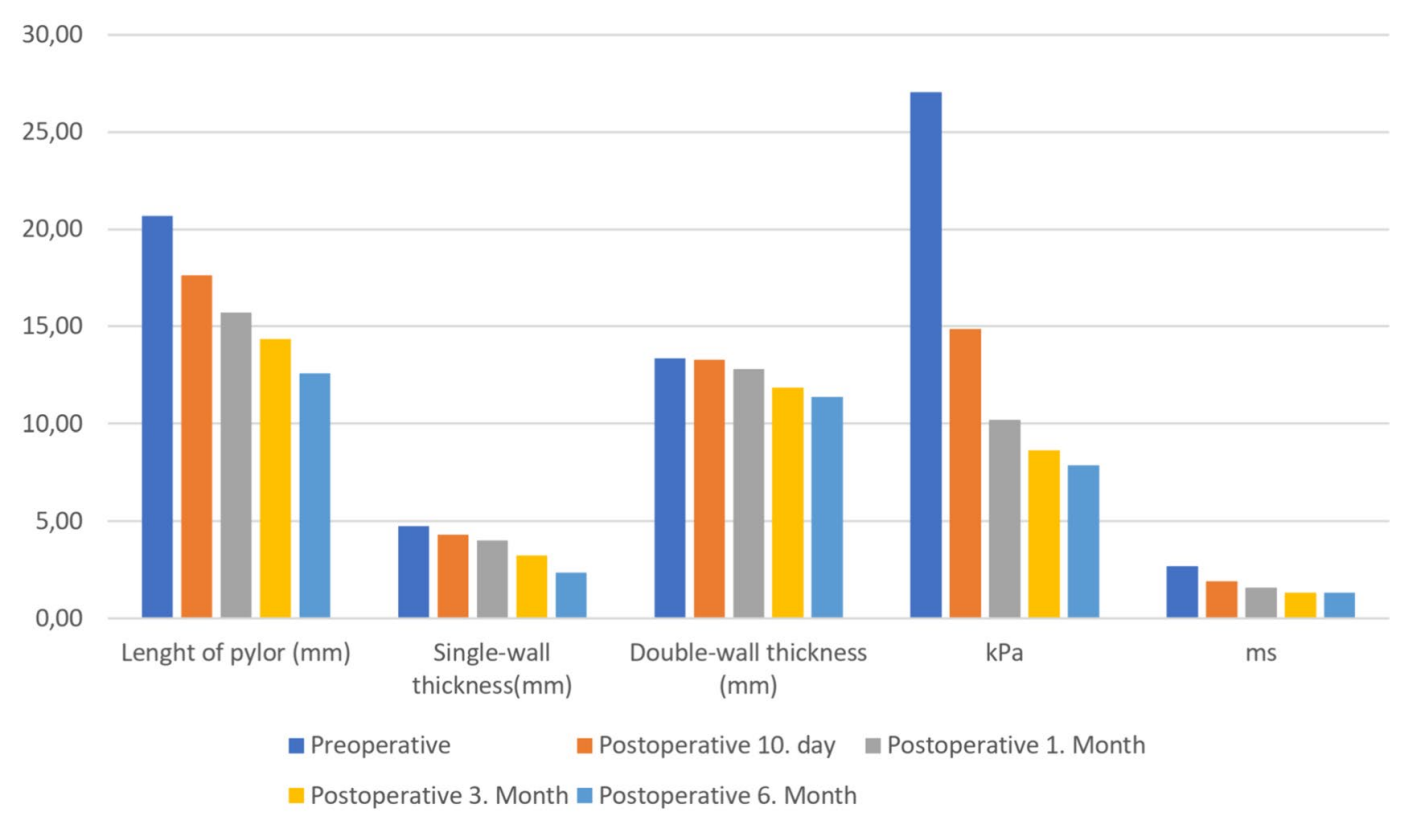
Changes over time in the lengths of the pylorus in B-Mode US and kPa and m/s values in 2D-SW-SE are shown

The patients were followed up at 10 days and at 1, 3, and 6 months postoperatively using B-mode US and 2D-SW-SE. Nine of the thirteen patients came to the follow-ups at 10 days and 1 month, but only five came to the follow-ups at 3 and 6 months, mostly due to restrictions resulting from the COVID-19 pandemic. Postoperative pyloric lengths, single-wall thicknesses, tissue stiffness, and propagation velocity measurements decreased gradually over the postoperative period, regressing to normal levels by the third month and remaining there at the sixth month. The values of postoperative follow-ups and changes in the values are detailed in Table 2 and Fig. 3.

## Discussion

Although B-mode US is the gold standard imaging method for diagnosing IHPS, its sensitivity and specificity are highly operator-dependent, and accurate diagnosis can therefore be difficult, especially in hospitals without experienced pediatric radiologists. Our study shows that, by measuring the elasticity of the pylorus, 2D-SWE-SE can be used as an assistive imaging tool alongside B-mode US for diagnosing IHPS with greater accuracy. It can also be used to diagnose IHPS by measuring the elasticity of the visible part of the pylorus in cases where the entire pylorus cannot be evaluated due to gas shadows in the intestinal tract or excessive agitation of the infant.

2D-SW-SE works by measuring the propagation speed of sound waves through tissues, with stiffer tissues resulting in higher speed because they have less elasticity and vice versa. Tissue stiffness is increased excessively by hypertrophy and hyperplasia in IHPS but gradually returns to normal after pyloromyotomy. In our study, the hardened pyloric tissues regressed to normal by the end of the third month in patients who underwent adequate surgical treatment, and a diagnosis of inadequate surgery can therefore be made more easily in patients whose 2D-SW-SE values do not return to normal by this point, making 2D-SW-SE a potentially more reliable imaging method than conventional B-mode US at follow-up.

USE is a non-invasive assisted ultrasound technology for evaluating the elasticity of tissues, and SW-USE has been used in many different fields of medicine [10, 13]. The liver is one of the organs most commonly examined with USE, particularly in the diagnosis and staging of liver diseases, especially liver fibrosis and liver tumors [15–17]. In children, USE has been used and found beneficial in determining whether fibrosis has developed following the treatment of biliary atresia with a Kasai operation and, if so, its stage [18, 19].

The muscles and skeletal system are also suitable structures for USE and SE evaluation because of their superficial location and elasticity, and many studies have therefore been conducted on musculoskeletal system diseases in adults [20–26]. In children, SE is considered beneficial in diagnosing and evaluating treatment responses to congenital muscular torticollis [27, 28]. Lee et al. reported that sternocleidomastoid muscles with congenital muscular torticollis have lower elasticity than normal muscle fibers [29].

Another field in which USE is used is the differential diagnosis of pathologies that cause lymphadenopathy. Most such studies have been conducted in adults [30]. Bhatia et al., stated that USE is useful in the differential diagnosis of cervical benign and malignant lymphadenopathies [31]. However, few studies have been done on children. One such study was by Bayramoğlu et al., who indicated that SW-USE is useful in diagnosing lymphoma in children and distinguishing it from lymphadenitis [32].

USE is also widely used with thyroid diseases [33, 34]; the elasticity of palpable and non-palpable nodules can be evaluated with USE, making it useful as an auxiliary test to differentiate benign from malignant thyroid tumors [35, 36]. Chronic kidney disease, renal fibrosis, renal tumors, diabetic renal disease, and urogenital, pancreatic, prostate, and spleen diseases can also be evaluated with USE techniques [11, 12, 37–40]. In a recent study, Fusco et al. claimed that USE could be used in the differential diagnosis of COVID-19 pneumoniae [41], and it has been reported that SW-USE has already been used in the diagnosis of other diseases associated with the COVID-19 pandemic [42].

There have been no studies to date on the use of 2D-SW-SE in the diagnosis of IHPS, so our study is the first in this area, but it has limitations. Firstly, it was conducted at a single center, and more meaningful results could be obtained if more than one center were involved. Nevertheless, we believe that our results will be supported by studies at different facilities. Secondly, our study had a small sample, with recruitment truncated by the COVID-19 pandemic. Because the approval period for the program expired after the pandemic, we did not have the chance to recruit more patients, and pandemic restrictions also created difficulties in following-up with patients.

## Conclusion

B-mode US remains the modality of choice for diagnosis and postoperative follow-up of IHPS, but 2D-SW-SE allows the stiffness of the tissue to be measured in addition to conventional B-mode US findings. This offers higher diagnostic accuracy, especially in cases where diagnosis is difficult for technical reasons, such as intestinal gas or agitation of the child, or because of insufficient radiologist experience. Furthermore, in infants whose vomiting continues during follow-up, an inadequate surgical diagnosis can be made more easily in cases where the elasticity of the pylorus, measured by 2D-SW-SE, does not decrease. We therefore recommend the use of 2D-SW-SE alongside conventional B-mode US in the diagnosis of IHPS and during postoperative follow-up, but multicenter studies with larger numbers of patients should be conducted to demonstrate the usability of the method.

## Data Availability

All data about the study are available in the corresponding author.
